# Pre-stroke adherence to a Mediterranean diet pattern is associated with lower acute ischemic stroke severity: a cross-sectional analysis of a prospective hospital-register study

**DOI:** 10.1186/s12883-020-01824-y

**Published:** 2020-06-23

**Authors:** Pablo M. Lavados, Enrico Mazzon, Alexis Rojo, Alejandro M. Brunser, Verónica V. Olavarría

**Affiliations:** 1 Departamento de Neurología y Psiquiatría, Clínica Alemana de Santiago, Facultad de Medicina, Clínica Alemana Universidad del Desarrollo, Unidad de Neurología Vascular, Servicio de Neurología, Santiago, Chile; 2 Departamento de Neurología y Psiquiatría y Departamento de Paciente Critico, Clínica Alemana de Santiago, Facultad de Medicina Clínica Alemana Universidad del Desarrollo, Unidad de Neurología Vascular, Servicio de Neurología, Avenida Vitacura 5951, Vitacura, 7650568 Santiago, Chile; 3 Servicio de Neurología, Hospital Clínico Herminda Martin de Chillán, Servicio de Salud Ñuble, Chillán, Chile; 4 Departamento de Neurología y Psiquiatría y Departamento de Urgencia, Clínica Alemana de Santiago, Facultad de Medicina, Universidad del Desarrollo, Clínica Alemana, Unidad de Neurología Vascular, Servicio de Neurología, Santiago, Chile

**Keywords:** Mediterranean diet, Adherence, Ischemic stroke, Acute stroke, Severity

## Abstract

**Background:**

High adherence to a Mediterranean Diet is associated with reduced incidence and mortality of acute ischemic stroke (AIS) but may also be associated with severity. Our purpose was to investigate the association of adherence to a Mediterranean diet and severity in a prospective hospital register of AIS patients.

**Methods:**

We included AIS patients admitted from February 2017 to July 2019. All were assessed by a neurologist with a standard stroke protocol, including NIHSS. Adherence to Mediterranean diet was prospectively measured by the 14-point Mediterranean Diet Adherence Screener (MEDAS) and defined as low (0–6 points) or high (7–14 points). Demographic and clinical characteristics were compared by group with univariate analysis. A Generalized Linear Model (GLM) was used to investigate the association of admission NIHSS as a continuous ordinal variable and an ordinal logistic regression (OLR) analysis to determine the independent association of the NIHSS quartiles with adherence to Mediterranean diet.

**Results:**

Three hundred sixty-eight patients were included, mean age 68.3 (17.7), 158 (42.9%) females. The median NIHSS score was 3 (IQR 1–9) and the median MEDAS score was 6 (IQR 4.5–8). Patients with high MEDAS scores had significantly lower; admission NIHSS scores, sedentary lifestyle, body mass index, total and LDL cholesterol levels, but higher alcohol consumption. After adjustments, high adherence to Mediterranean diet remained independently associated with lower stroke severity both in the GLM (β coefficient = − 0.19, *p* = 0.01) and in the OLR model (OR for lower NIHSS quartiles 0.6 (95% CI 0.37–0.98, *p* = 0.04).

**Conclusions:**

Higher pre-stroke adherence to a Mediterranean diet is independently associated with lower AIS severity.

## Background

Eating patterns and their components may be predictive of health status and disease risk factors. According to the INTERSTROKE study, the population attributable risk of unhealthy diet on stroke was 19% (11–30%) [1, 2]. Furthermore the Global Burden of Disease demonstrated that in 2013, 63.4% (56.5–67.8) of disability-adjusted life years (DALYs) due to stroke could be attributed to dietary risks [3]. Adherence to dietary patterns such as the Dietary Approaches to Stop Hypertension (DASH), the Nordic and the Mediterranean diets have shown to decrease both incidence and mortality of stroke in general [4]. High adherence to a Mediterranean Diet pattern is associated with reduced incidence of acute ischemic stroke (AIS) and mortality both in observational as well as in a large primary prevention clinical trial [5, 6]. The beneficial role of this diet could be through modifying classical cardiovascular risk factor such as high blood pressure, diabetes, obesity and the metabolic syndrome [7–9].

A Mediterranean diet may also be associated with less severity at onset and better prognosis of ischemic stroke. This has only been investigated in a small retrospective study which reported that in 198 patients with AIS, those with lower adherence to a Mediterranean diet defined according to a food frequency questionnaire, were more likely to have a large artery atherothrombotic etiology, a worse clinical presentation at admission and a worse prognosis at discharge [10].

Our aim was to investigate the association of the adherence to the Mediterranean diet with stroke severity in a prospective hospital-based register of patients with AIS.

## Methods

### Study population

Our institution is a teaching non-for-profit tertiary private hospital of Santiago, Chile. Since 1997 we include all consecutive 18 year or older patients with an acute stroke admitted to Clínica Alemana de Santiago in our prospective stroke registry (Registro de Enfermedades Cerebrovasculares Clínica Alemana: RECCA).

In this study we selected patients with AIS, complete data on the 14-point Mediterranean Diet Adherence Screener (MEDAS) score and informed consent, admitted from February 2017 to July 2019. We excluded patients with transient ischemic attacks.

All variables were prospectively collected during patient hospitalization. Ischemic stroke was defined according to current practice [11]. Ischemic stroke etiology was classified using the Trial of Org 10,172 in Acute Stroke Treatment (TOAST) classification [12]. Hypertension, Diabetes Mellitus, Insulin resistance, hyperlipidemia and mood disorder were defined as present in patients with the previous clinical diagnosis or in those under treatment for each specific risk factor. Vessel occlusion was defined present when found in intracranial o cervical extracranial arteries on acute vascular neuroimaging as per institutional protocol. Previous dependency was defined according to the modified Rankin Scale (mRS). Patients with suspected stroke were immediately evaluated by the neurologist. Acute neurological assessment includes: the National Institutes of Health Stroke Scale (NIHSS) score [13] and a neuroimaging stroke protocol consisting of: non-contrast Computed Tomography (CT) scan, Diffusion Weighted Magnetic Resonance Imaging (DWI-MRI) and cervico-craneal CT angiography (CTA) or magnetic resonance angiography (MRA). Additionally, many patients are studied with Transcranial Doppler and some Digital Subtraction Angiography (DSA). Etiologic evaluation includes electrocardiogram monitoring, Echocardiography and Carotid Ultrasonography if appropriate as well as coagulation, hematological and biochemical analyses.

Adherence to a Mediterranean diet was measured by the Spanish version of the MEDAS score, which we adapted to the local Chilean terms for an ongoing population-based stroke study [14]. The MEDAS is a 14-point questionnaire validated and used in the PREDIMED study to assess the adherence to a Mediterranean diet pattern (supplement); higher scores are associated with higher adherence, being 14 points the maximum score [15]. We used this short screener as it has been shown to be a valid tool for rapid assessment of adherence to Mediterranean diet pattern and proposed as useful in time-limited clinical and research settings [16]. Since January 2017, the stroke fellow or vascular neurology staff obtains the MEDAS score prospectively in all patients admitted with an AIS diagnosis, using a paper form previous to discharge in one session. Exposure before the index stroke was assessed by asking the patients (85%) or next of kin (15%) to refer to their last month usual diet pattern. Adherence to Mediterranean diet pattern was defined as low (score of 0–6 points) or high (score of 7–14 points) based on the median MEDAS scores in our cohort. The primary outcome was severity defined by the admission NIHSS score.

The local Ethics Committee and the Institutional Review Board of the hospital approved the study registry protocol and written informed consent was obtained in every patient as local regulatory law requests.

### Statistical analyses

Sociodemographic characteristics, cardiovascular risk factors, medication use, clinical presentation, etiology, treatments and NIHSS scores were compared by adherence to the MEDAS score using Chi^2^ or Fisher’s test for frequencies. T-test was used for normally distributed continuous variables and Wilcoxon log-rank test for non-normally distributed continuous variables in the univariate analyses.

We investigated the association of adherence to Mediterranean diet and NIHSS scores computed as an ordinal variable using a Generalized Linear Model (GLM) with gamma distribution and link function identity adjusting for the following explanatory variables: Diabetes Mellitus, sedentary lifestyle, alcohol consumption, Body Mass Index (BMI), total and Low-Density Lipoprotein (LDL) cholesterol. These were chosen because their frequency distribution was significantly different in both groups in univariate comparison. In this model we further adjusted for prognostic variables usually associated with severity in prior studies, using Hosmer–Lemeshow criteria with a cut-off point of *p* < 0.25 [17]. These prognostic variables were age, previous dependency (mRS 3–5), atrial fibrillation (AF), sedentary lifestyle, time from symptom onset to emergency consultation, vessel occlusion, admission glycemia, and cardioembolic etiology. In order to decrease over-adjustments, we only included cardioembolic stroke and not atrial fibrillation in the models [18–20]. In this model we added 1 to all NIHSS scores, because 62 (16.8%) patients had a cerebral infarction but an NIHSS of 0. In order to account for the effect of premorbid disability on severity we performed a sensitivity analysis stratifying by previous disability, excluding those with mRS 3–5 from the GLM model.

As a secondary analysis we performed an ordinal logistic regression (OLR) to determine the independent association of low or high adherence to Mediterranean diet to quartiles of NIHSS scores. NIHSS quartiles are: 0–1 = q1; 2–3 = q2; 4–9 = q3; 10-maximum = q4. The prognostic and confounding variables in the ORL model were the same as in the previous model: Age, Diabetes mellitus, alcohol consumption, sedentary lifestyle, previous dependency, time from symptom onset to hospital admission, vessel occlusion, body mass index, admission glucose, total cholesterol, LDL cholesterol and cardioembolic stroke etiology.

All statistical analyses were performed with Stata 14.0. An alfa error < 0.05 was considered significant. The paper is reported according to the STROBE guidelines [21].

## Results

**Fig. 1:**
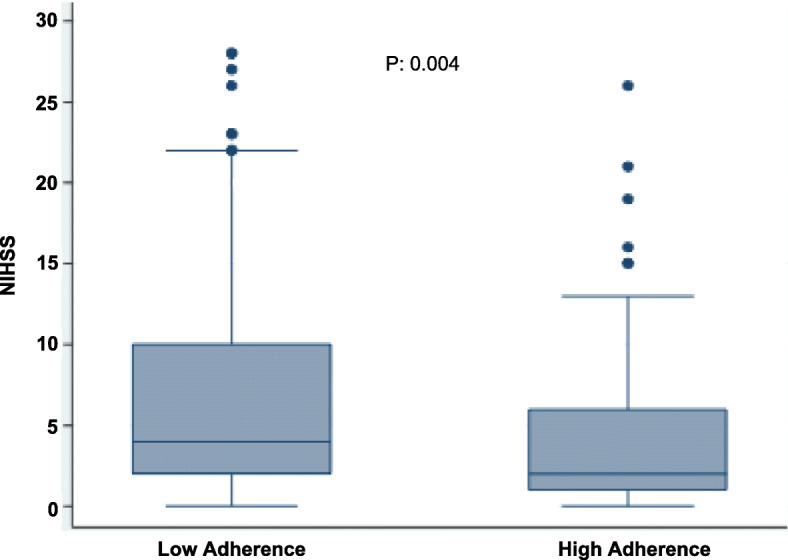
Admission NIHSS scores according to high or low adherence to Mediterranean diet

From February 2017 to July 2019, 444 patients with AIS and complete MEDAS scores were admitted, of which 76 transient ischemic attacks were excluded. The study sample thus consisted of 368 patients with a mean age of 68.3 (17.7) years and 158 (42.9%) being female. The median time from symptom onset to admission was 228 min (IQR 64–783). The median admission NIHSS score was 3 (IQR 1–9) and the median MEDAS score was 6 (IQR 4.5–8). High adherence to Mediterranean diet pattern was found in 151 (41%) patients. Table 1 describes the baseline demographic, clinical and laboratory characteristics of both groups. Patients with high MEDAS scores had significantly lower admission NIHSS scores with a median of 2 (IQR 2–6) versus a median 4 (IQR 2–10, *p* = 0.004) (Fig. 1), and a lower frequency of: Diabetes Mellitus, a less sedentary lifestyle and lower BMI but higher frequency of alcohol consumption. Patients with higher adherence also had significantly lower total and LDL cholesterol levels.

**Table 1:** Baseline characteristics of ischemic stroke patients according to adherence to a Mediterranean diet using MEDAS score

Variable	High adherence N (%) 151 (41)	Low adherence N (%) 217 (59)	Total N (%) 368 (100)	*P* Value
Age, years, mean (SD^a^)	69.9 (16.9)	67.2 (18.7)	68.3 (17.7)	0.1
Gender (female)	66 (43.7)	92 (42.4)	158 (42.9)	0.8
Hypertension	89 (58.9)	133 (61.3)	222 (60.3)	0.5
Diabetes Mellitus	17 (11.2)	41 (18.9)	58 (15.7)	0.04
Insulin resistance	15 (9.9)	17 (7.8)	32 (8.7)	0.4
Dyslipidemia	56 (37.0)	67 (30.8)	123 (33.4)	0.2
Coronary artery disease	20 (13.2)	28 (9.21)	48 (13.0)	0.7
Atrial fibrillation	26 (17.2)	40 (18.4)	66 (17.9)	0.7
Prior stroke	27 (17.9)	36 (16.6)	63 (17.1)	0.7
Chronic Renal Failure	8 (5.3)	21 (9.7)	29 (7.9)	0.1
Mood disorder	19 (12.6)	32 (14.7)	51 (13.8)	0.5
Current smoker	34 (22.5)	46 (21.2)	80 (21.7)	0.2
Alcohol consumption	76 (50.3)	83 (38.4)	159 (43.3)	0.02
Sedentary lifestyle	90 (59.6)	172 (79.3)	262 (71.2)	< 0.001
Previous dependency^b^	11 (7.3)	29 (13.4)	40 (10.9)	0.06
Time from symptom onset to admission, median (IQR^c^)	165 (72–706)	248 (78–1385)	228 (68–784)	0.03
Admission Systolic Blood Pressure, mean (SD)	148 (25.0)	151 (26.2)	150 (25.7)	0.1
Admission Diastolic Blood Pressure, mean (SD)	80.9 (15.1)	83.3 (17.6)	83.3 (16.6)	0.1
Admission body mass index, Mean (SD)	25.1 (3.4)	26.7 (4.4)	26.0 (4.1)	0.001
Admission NIHSS^d^ median (IQR)^c^	2-(1-6)	4 (2-11)	3 (1-7)	0.0006
Vessel occlusion	45 (29.8)	72 (33.2)	117 (31.8)	0.5
Intravenous thrombolysis use	53 (35.19	78 (35.9)	131 (35.6)	0.8
Intra-arterial thrombectomy	18 (11.9)	20 (9.0)	38 (10.3)	0.4
Large-artery atherosclerosis	29 (19.1)	47 (21.7)	76 (20.7)	0.5
Cardioembolic	48 (31.7)	62 (28.7)	110 (29.29)	0.5
Dissection	11 (7.2)	18 (8.3)	29 (7.9)	0.7
Other determined	10 (6.2)	14 (6.4)	24 (6.5)	0.9
Cryptogenic (complete work-up)	48 (31.8)	52 (23.9)	100 (27.1)	0.09
Undetermined (incomplete work-up)	3 (1.9)	6 (2.7)	9 (2.4)	0.6
Not classifiable (2 or more causes)	4 (2.6)	14 (6.4)	18 (4.9)	0.09
Glucose, (mg/dl), mean	117.3 (35.6)	124.9 (47.6)	121.7 (43.1)	0.07
Total cholesterol, (mg/dl), mean	157.3 (45.8)	169.2 (47.4)	164.1 (46.9)	0.02
LDL^e^ cholesterol, (mg/dl), mean	99.2 (38.8)	110.9 (43.4)	105.9 (42.1)	0.01
HDL^f^ cholesterol, (mg/dl), mean	48.6 (14.8)	47.2 (15.2)	48.1 (15.0)	0.6
Triglycerides, (mg/dl), mean	130.7 (111.9)	138.4 (82.6)	135.1 (96.3)	0.2
HbA1c^g^ (%), median (IQR^c^)	5.6 (5.3–6.0)	5.6 (5.3–6.0)	5.6 (5.3–6.0)	0.8
Hemoglobin, (g/dl), mean	13.9 (1.7)	13.7 (2.1)	13.8 (1.9)	0.5
Leucocyte count	2639 (4479)	2643 (4439)	2641 (4449)	0.9

^a^*SD* standard deviation, ^b^*mRS* (modified Rankin Scale) = 3–5, ^c^*IQR* interquartile range, ^d^*NIHSS* National Institutes of Health Stroke Scale, ^e^*LDL* Low-Density Lipoprotein, ^f^*HDL* High-density Lipoprotein, ^g^*HbA1c* glycosylated haemoglobin A1c

The GLM showed an inverse independent association of adherence to Mediterranean Diet with NIHSS scores (β coefficient = − 0.19, *p* = 0.01); indicating that for every unit increase of MEDAS score, we estimate a decrease of 0.2 points of admission NIHSS scores (Table 2). The sensitivity analysis showed that the association between higher adherence to a Mediterranean diet and stroke severity was significant after excluding patients with previous disabilities from the analysis (β coefficient = − 0.18, *p* = 0.02).

**Table 2:** Adjusted Gamma regression model of adherence to Mediterranean diet and admission NIHSS^b^ scores

Variable	Adjusted Coefficient^a^	95% Confidence interval		*P* value
High adherence to Mediterranean diet^c^	−0.1995912	−0.3625112	−0.0364712	0.016
Age	0.0255986	0.0004482	0.0507489	0.046
Longer time from symptom onset to admission	−0.0000643	− 0.0000981	−0.0000304	< 0.0001
Cardioembolic etiology	1.581582	0.263316	2.899848	0.019 0.043
Diabetes Mellitus	1.833642	0.0329643	3.63432	0.046
Vessel occlusion	6.217865	4.314902	8.120828	< 0.0001

^a^Adjusted for: Age, Diabetes Mellitus, alcohol consumption, sedentary lifestyle, previous dependency, time from symptom onset to hospital admission, vessel occlusion, body mass index, admission glucose, total cholesterol, LDL cholesterol, cardioembolic stroke etiologies. ^b^*NIHSS* National Institutes of Health Stroke Scale. ^c^High adherence to Mediterranean diet = MEDAS scores 7–14

The results of the OLR model are presented in Table 3. A high adherence to Mediterranean diet was protective of stroke severity resulting in an Odds Ratio for higher quartiles of NIHSS of 0.6 (95% CI 0.37–0.98, *p* = 0.04).

**Table 3:** Adjusted^a^ ordinal logistic regression model of adherence to a Mediterranean diet on increasing NIHSS admission quartiles^b^

Variable	Odds Ratio	95% Confidence interval		*P* value
High adherence to Mediterranean diet^c^	0.602	0.369	0.9828	0.042
Symptom onset to admission	0.9999	0.9998	0.9999	0.001
Cardioembolic etiology	1.963084	1.165307	3.307026	0.011
Previous dependency	4.063095	1.552344	10.63472	0.004
Diabetes Mellitus	2.029745	1.009952	4.079267	0.047
Alcohol consumption	0.5730412	0.3581713	0.9168132	0.02
Vessel occlusion	6.253156	3.650669	10.7109	< 0.0001

^a^Adjusted for: Age, Diabetes Mellitus, alcohol consumption, sedentary lifestyle, previous dependency, time from symptom onset to hospital admission, vessel occlusion, body mass index, admission glucose, total cholesterol, LDL cholesterol, cardioembolic stroke etiology

^b^NIHSS quartiles are 0–1 = q1; 2–3 = q2; 4–9 = q3; 10-maximum = q4

^c^High adherence to Mediterranean diet = MEDAS scores 0–6

## Discussion

In this cross-sectional study we found that a high previous adherence to Mediterranean Diet was independently associated with lower ischemic stroke severity on admission. These results are consistent with a previous retrospective study, which reported a worse outcome by means of higher NIHSS in patients with low adherence to a Mediterranean diet pattern [7]. Patients with higher adherence in this cohort smoked less, had less Diabetes Mellitus, lower BMI, were less sedentary and had lower total and LDL cholesterol levels. This has been described earlier and could reflect not only an overall healthier lifestyle, but also a lower prevalence of the metabolic syndrome [8, 22–26]. A combination of good healthy lifestyle could have a greater impact that each factor alone. In fact combination of four healthy behaviors (current non-smoking, physically active, moderate alcohol consumption, and at least 5 serving a day of fruit and vegetables) was found to have a higher impact in the magnitude of the incidence of stroke in men and women aged 40–79, with a dose response association predicting more than double difference in the incidence compared to controls after adjustments [27]. Furthermore, the combination of eating a healthy diet, exercising regularly, maintaining a low BMI and drinking alcohol in moderation was independently associated with all-cause and cardiovascular mortality following a stroke, after adjustments [28].

In this cohort of AIS patients, the mean MEDAS score was 6 (SD 2.3) points, which is very similar to the mean 5.7 (SD 1.6) found in 53,366 adults in a validation study of a self-applicable questionnaire for a Mediterranean dietary index in Chile and higher than that found in an intervention study in 96 workers in Chile whom had a basal score of 4.8 (SD 1.4). In the latter, the Mediterranean score increased to 7.4 (SD 1.5) after a non-randomized dietary intervention in the workers canteen, resulting in a reduction of the prevalence of the metabolic syndrome from 24.0 to 15.6% (*p* = 0.02) [29, 30].

A recent Cochrane review shows that Mediterranean diet reduces the incidence of stroke from 24/1000 to 14/1000 (95% CI 11 to 19) and this could be mediated by reductions in blood pressure and possible small reductions in blood lipids [31]. Other proposed mechanisms are the effects of the Mediterranean diet on systemic inflammation, which could have a role in acute inflammatory activation, improved endothelial health, decreasing intestinal bio-microbiome mediated atherothrombosis and toxicity, and by increasing plasma redox status [32–36].

In stroke patients there is evidence of the association of prognosis and inflammatory biomarkers [33]. Similar results have been shown in high cardiovascular risk populations, in particular hypertension, diabetes and the metabolic syndrome [37]. The effect of the Mediterranean diet on stroke severity could be mediated by a lower inflammatory response as suggested by less pro-inflammatory biomarker activation in these patients. This in turn could promote different pathogenic stroke mechanisms [10, 33]. In fact in our study there were no differences regarding atherothrombotic etiology as previously reported, [10, 38] but we did observe a non-significant higher frequency of cryptogenic stroke in patients with higher adherence to a Mediterranean diet, maybe suggesting a predominance of stroke mechanisms less associated to classical cardiometabolic cardiovascular risk factors and less severe strokes [39].

Strengths of this study are that all data on exposures was collected prospectively, particularly the MEDAS scores and that the NIHSS scores have been shown to be highly reproducible [13].

Limitations of this observational study are the sample size and being a single hospital cohort limiting external validity. The possibility of recall bias is another limitation, but we have no indication that patients with high or low NIHSS would have a differential recall of their usual previous diet patterns. Another caveat is that even though the NIHSS is widely used to assess stroke severity at the bedside it has not been extensively validated for this purpose as it was developed for use mainly in clinical trials [13]. An additional consideration is that we chose the MEDAS score for its practicality and international validation, but there are another 28 scores evaluating the degree of adherence to Mediterranean diet patterns [16, 40]. A recent systematic review suggested that 3 of these were the ones showing the highest level of evidence of conceptual suitability, applicability and psychometric properties [41]. Furthermore we did not use the Chilean version of Mediterranean diet (Chilean MDI) validated as self-administered questionnaire because we administered the score ourselves [29, 30]. Another possible source of bias is that we obtained data on dietary intake of next-of- kin in approximately 15% of the patients. Previous studies have described a high agreement between mean intakes of specific foods reported by interviewing subjects and their surrogates, implying that surrogate data may be useful in descriptive studies, providing an unbiased estimate of mean consumption by a group, but these studies have included a limited number of elderly subjects and were dependent of the frequency of food consumption, gender of the index subject, the relationship of the surrogate to the index subject (being higher for spouses), and the method of data collection [42].

Our findings suggest that high adherence to a Mediterranean diet defined by the MEDAS score seems to have benefits not only in reducing stroke incidence as previously shown but also in decreasing stroke severity which could have an impact on long term disability and the global burden of stroke [43]. Changing dietary habits and improving adherence to a Mediterranean diet has been shown to decrease cardiometabolic risk factors and stroke in selected or high risk populations, but it is not clear if this strategy could be used at a population level [4, 6, 30, 31].

## Conclusion

In this observational prospective study higher adherence to a Mediterranean Diet was associated with less severe acute ischemic stroke at admission. Further larger studies should confirm this finding and clarify if it has an impact in long term functional outcome.

## Availability of data and materials

The datasets used and/or analyzed during the current study available from the corresponding author on reasonable request.

## Supplementary information

**Additional file 1:.** Microsoft word file. 14-point Mediterranean Diet Adherence Screener (MEDAS).

**Additional file 2:.** Excel spread sheet 1997–2003 (.xls). RECCA2015_DATA_LABELS_2019-07-17_1307 MeDi y NIHSS Febrero 2017 Julio 2019. Dataset supporting the conclusions.

## Data Availability

The dataset supporting the conclusions of this article is included within the article as an additional Excel file.

## Abbreviations

AIS: Acute ischemic stroke
MEDAS: Mediterranean Diet Adherence Screener
GLM: Generalized Linear Model
OLR: Ordinal logistic regression
DALYs: Disability-adjusted life years
TOAST: Trial of Org 10,172 in Acute Stroke Treatment
RECCA: Registro de Enfermedades Cerebrovasculares Clínica Alemana
mRS: modified Rankin Scale
NIHSS: National Institutes of Health Stroke Scale
CT: Computed Tomography
DWI-MRI: Diffusion Weighted Magnetic Resonance Imaging
CTA: Cervico-craneal CT angiography
MRA: Magnetic resonance angiography
DSA: Digital Subtraction Angiography
BMI: Body Mass Index
LDL: Low-Density Lipoprotein
Chilean MDI: Chilean version of Mediterranean diet
