# Supplementary material for: Pre-stroke adherence to a Mediterranean diet pattern is associated with lower acute ischemic stroke severity: a cross-sectional analysis of a prospective hospital-register study
Source: BMC Neurol. 2020 Jun 23;20:252. doi: 10.1186/s12883-020-01824-y (PMC7310482; doi:10.1186/s12883-020-01824-y)
Supplement: Supplementary file 1 — Additional file 1:. Microsoft word file. 14-point Mediterranean Diet Adherence Screener (MEDAS). [file 12883_2020_1824_MOESM1_ESM.docx]

Additional file 1.

14-point Mediterranean Diet Adherence Screener (MEDAS)

| **Item** | **Criteria for obtaining 1 point** |
| --- | --- |
| **1. Do you use olive oil as the principal source of fat for cooking?**  ¿Usa usted el aceite de oliva como principal grasa para cocinar? | Yes = 1 point |
| **2. How much olive oil do you consume per day (including that used in frying, meals eaten away from home, salads, etc.)? (1 tablespoon = 13.5g).**  ¿Cuánto aceite de oliva consume en total al día (incluyendo el usado para freír, comidas fuera de casa, ensaladas, etc.)? | 4 or more  tablespoons = 1 point |
| **3. How many servings of vegetables do you consume per day? (1 serving = 200g - side dishes count as ½ a serving, not including potatoes or sweetcorn).**  ¿Cuántas porciones de verduras u hortalizas consume al día? (las porciones o acompañamientos equivalen a 1/2 ración) 1 porción = 200g. | 2 or more (at  least 1 portion  raw or as salad) = 1 point |
| **4. How many pieces of fruit (including fresh-squeezed fruit juice) do you consume per day? (not including frozen or dried fruit).**  ¿Cuántas unidades de fruta (incluyendo las que utiliza para hacer jugo natural) consume al día? | 3 or more = 1 point |
| **5. How many servings of red meat, hamburger, or meat products (ham, sausage, etc.) do you consume per day? (1 serving = 100-150g)**  ¿Cuántas porciones de carnes rojas, hamburguesas, salchichas o embutidos consume al día? (porción: 100 - 150 g equivalente a la palma de la mano o 1 unidad de hamburguesa o vienesa) | Less than 1= 1 point |
| **6. How many servings of butter, margarine, or cream do you consume per day? (1 serving = 12g, 1 tablespoon).**  ¿Cuántas porciones de mantequilla, margarina o crema consume al día? (considerando una porción individual equivalente a 1 cucharada de sopera o 12 g) | Less than 1= 1 point |
| **7. How many sugar-sweetened beverages do you drink per day? (1 cup = 100 ml).**  ¿Cuántas bebidas carbonatadas y/o azucaradas (como refrescos, bebidas colas, tónicas) consume al día? | Less than 1 cup = 1 point |
| **8. How much wine do you drink per week? (1 glass = 125 ml)**  ¿Bebe usted vino? ¿Cuánto consume a la semana? | 7 or more  Glasses = 1 point |
| **9. How many servings of legumes do you consume per week? (1 serving = 150g) (including canned varieties).**  ¿Cuántas porciones de legumbres consume a la semana? (una porción equivale a un plato hondo o 150 g) | 3 or more = 1 point |
| **10. How many servings of fish or shellfish/seafood do you consume per week? *(1 serving = 100-150 g fish, or 4-5 pieces or 200 g shellfish).***  ¿Cuántas porciones de pescado o mariscos consume a la semana? (1 plato pieza o ración equivale a 1 palma de mano de pescado (100 – 150g) o 4-5 piezas de marisco(200g) ) | 3 or more = 1 point |
| **11. How many times per week do you consume commercial sweets or pastries (not homemade), such as cakes, cookies, biscuits, or custard?** ¿Cuántas veces consume productos de repostería comercial (no casera) como galletas, flanes, dulce o pasteles a la semana? | 2 or less = 1 point |
| **12. How many servings of nuts (including peanuts) do**  **you consume per week? (1 serving = 30g).**  ¿Cuántas veces consume frutos secos a la semana? Como nueces, almendras, maní, pistachos (una ración equivale a un puño de mano cerrada o 30 g) | 3 or more = 1 point |
| 13. **Do you prefer to eat chicken, turkey, or rabbit meat instead of beef, pork, hamburgers, or sausages?**  ¿Consume usted preferentemente carne de pollo, pavo en vez de vacuno, cerdo, hamburguesas o salchichas? (carne de pollo: 1 pieza o ración de 100 - 150 g) | Yes = 1 point |
| **14. How many times per week do you consume cooked vegetables, pasta, rice, or other dishes prepared with a sauce of tomato, garlic, onions or leeks sautéed in olive oil (sofrito)?**  ¿Cuántas veces a la semana consume vegetales, pasta, arroz u otros platos cocinados con un sofrito (de salsa de tomate, ajo, cebolla o puerro elaborada a fuego lento con aceite de oliva)? | 2 or more = 1 point |
| Total score: |  |
